# Clinical and pathological features of five-year survivors after pancreatectomy for pancreatic adenocarcinoma

**DOI:** 10.1186/1477-7819-12-360

**Published:** 2014-11-27

**Authors:** Kenjiro Kimura, Ryosuke Amano, Bunzo Nakata, Sadaaki Yamazoe, Keiichiro Hirata, Akihiro Murata, Kotaro Miura, Kohei Nishio, Toshiki Hirakawa, Masaichi Ohira, Kosei Hirakawa

**Affiliations:** Department of Surgical Oncology, Osaka City University Graduate School of Medicine, 1-4-3 Asahimachi, Abeno-ku, Osaka, 545-8585 Japan; Department of Surgery, Kashiwara Municipal Hospital, 1-7-9 Hozenji, Kashiwara City, Osaka, 582-0005 Japan

**Keywords:** Pancreatic carcinoma, Five-year survivors, CA19-9, R0, Lymph node metastasis

## Abstract

**Background:**

Clinical factors determining short-term survival after pancreatectomy have been well studied, but factors predicting long-term survival with curative resection are poorly understood in pancreatic carcinoma. Our objective was to identify clinical and pathological features of five-year disease-free survivors after surgical resection of pancreatic adenocarcinoma.

**Methods:**

The clinical and pathological data from 147 patients who underwent a potentially curative resection for pancreatic adenocarcinoma at our institution between 1988 and 2012 were retrospectively analyzed.

**Results:**

Of 147 patients, 18 survived for more than five years after surgery without disease recurrence. A univariate analyses demonstrated that: two or fewer lymph node metastases (*P* = 0.014), a preoperative serum carbohydrate antigen 19-9 (CA19-9) level of 40 U/mL or less (*P* = 0.0018), an absence of intrapancreatic nerve invasion (*P* = 0.028), and undergoing an R0 resection (*P* = 0.011) were significantly associated with five-year survival. A logistic regression model identified the following independent cancer-related predictors of five-year survivors: having two or fewer lymph node metastases (odds ratio (OR): 6.02; 95% confidence interval (CI): 1.08 to 112.98; *P* = 0.0385), a preoperative serum CA19-9 level of 40 U/mL or less (OR: 5.02; 95% CI: 1.68 to 16.48; *P* = 0.0036), and undergoing an R0 resection (OR: 3.63; 95% CI: 1.12 to 14.28; *P* = 0.0316).

**Conclusions:**

We conclude that number of lymph node metastases being two or less, a preoperative serum CA19-9 level of 40 U/mL or less, and undergoing an R0 resection may be independent predictive factors to identify actual five-year survivors after pancreatectomy for pancreatic adenocarcinoma.

## Background

Pancreatic carcinoma is the fourth leading cause of death from cancer and is responsible for 43,000 deaths per year in the United States [1]. The prevalence of pancreatic cancer in Japan has also increased in the last decade to become the fifth leading cause of cancer death in men, and the sixth in women [2]. This malignancy is devastating, with a five-year overall survival rate of approximately 5% [1]. The only potentially curative treatment for pancreatic cancer is surgical resection. However, only a small number of patients (between 15 and 20%) present with a resectable tumor at the time of diagnosis [3]. Moreover, the prognosis even after potentially curative resection is considered to be poor. The following characteristics have been reported to be significant prognostic factors for patient survival after tumor resection: age [4], tumor size [4–6], lymph node metastasis [4–6], surgical margin status [7–9], preoperative serum CA19-9 level [9–11], and tumor grade [7]. Clinical factors determining short-term survival after pancreatectomy have been well studied, but prognostic factors predicting long-term survival with curative resection are poorly understood [12–14].

In the earlier studies, it was difficult to discuss factors related to five-year survival because of the high postoperative mortality. Today, the surgical procedure can be performed safely, and the postoperative mortality in some specialized pancreatic centers is reported to be less than 5% [15–17]. In the many previous reports, the follow-up period was within five years, but precise data on the long-term survival and prognostic factors can be obtained by analysis not only of actuarial data, but also of data of patients who achieve actual long-term survival of five years or more.

The aim of this study was to identify the clinical and pathological features of five-year survivors after surgical resection of pancreatic ductal carcinoma. This study could aid oncologists and surgeons in determining which characteristics or clinicopathological factors suggest an increased possibility of five-year survival after pancreatic resection for pancreatic carcinoma.

## Methods

### Patients

A total of 195 patients who underwent pancreatectomy for pancreatic ductal carcinoma at our institution between January 1988 and October 2012 were studied. Informed consent was obtained from all patients to use the specimens for this study according to the institutional rules of the hospital. All patients were histologically confirmed to have the common type of invasive ductal carcinoma of the pancreas. Any patients with neuroendocrine carcinoma, mucinous cystic carcinomas, or intraductal papillary mucinous carcinomas were excluded. Of the 195 patients, 48 patients were excluded for the following reasons: 42 censored cases, composed of four patients who were lost to follow-up during the observation period and 38 patients who were alive within five years after the operation; four due to postoperative mortality within 30 days; and two were five-year survivors with recurrence disease. The data from the remaining 147 patients, who were five-year survivors without disease recurrence (five-year survivors) and died within five years after surgery (short-term survivors), was retrospectively analyzed. The demographic and clinical variables included age, sex, preoperative serum CA19-9 level, and tumor location. In patients with preoperative jaundice, the data after the jaundice was reduced was used as the preoperative serum CA19-9 values. In patients with jaundice at our medical center, endoscopic or percutaneous bile duct drainage is usually performed. The CA19-9 value in all patients was the value after total bilirubin was reduced to under 5 mg/dL. All patients had presented with resectable localized disease without distant metastasis. None of the patients received neoadjuvant therapy before surgery. Fourteen patients had intraoperative radiation therapy (IORT), and 20 patients received adjuvant chemotherapy with gemcitabine. All patients were followed up on for survival, and the median follow-up period was 14.5 (2.1 to 170.2) months.

### Surgery and pathology

Surgery involved standard or subtotal stomach-preserving pancreaticoduodenectomy in 90 patients (61.2%), distal pancreatectomy in 49 (33.3%), and total pancreatectomy in eight (5.4%). Regional lymph node dissection was performed in all patients and the median number of resected lymph nodes was 24 (range: 2 to 100). The resected specimens were fixed in 10% formalin at room temperature, and the size and gross appearance of the tumor were recorded. The pathologic stage of all tumor specimens was determined using the American Joint Committee on Cancer (Sixth edition) staging system [18]. Tumor differentiation was classified according to the World Health Organization’s classification of either well-differentiated (Grade 1), moderately differentiated (Grade 2), poorly differentiated (Grade 3), or undifferentiated (Grade 4) [19]. A positive margin was defined as the presence of at least one cancer cell within 1 mm of one or more resection margins on a macroscopic examination. The pathological features that might affect prognosis were histologically assessed tumor size, serosal invasion (S), retroperitoneal tissue invasion (RP), intrapancreatic common bile duct invasion (CH), portal vein invasion (PV), lymph node metastasis, lymphatic invasion (LY), venous invasion (V), and intrapancreatic nerve invasion (NE), on the basis of the Japan Pancreas Society classification (Sixth edition) [20].

### Statistical analysis

The clinicopathological features were compared between five-year survivors and short-term survivors. The risk factors related to survival were examined in long-term survivors. Categorical variables were compared using the *χ*^2^ test or Fisher’s exact test. A receiver operating characteristics (ROC) curve was constructed to estimate the optimal cutoff value of preoperative serum CA19-9. The cutoff value was determined as the point closest to the upper left-hand corner of the graph. Variables with a significance of *P* <0.05 on a univariate analysis were included in a multivariate regression analysis to identify factors associated with long-term survival. Survival was calculated using the Kaplan-Meier method and compared between groups by the log-rank test. *P* values <0.05 were considered significant. Statistical analyses were performed using SAS version 9.0 software (SAS Institute, Inc., Cary, North Carolina, United States).

## Results

Characteristics of patient and tumor-related data of these 147 patients are given in Table 1. The median overall survival of all cases was 14.4 months; short-term and five-year survivors were 12 months and 125.6 months, respectively. The actuarial three- and five-year survival rates were 18.4% and 12.2%, respectively. The median overall survival times of early cases (who were operated on between 19888 and 2000) and late cases (2001 to 2012) were 13.5 and 14.7 months, respectively. There are no statistical differences in survival (*P* = 0.65).

**Figure 1:**
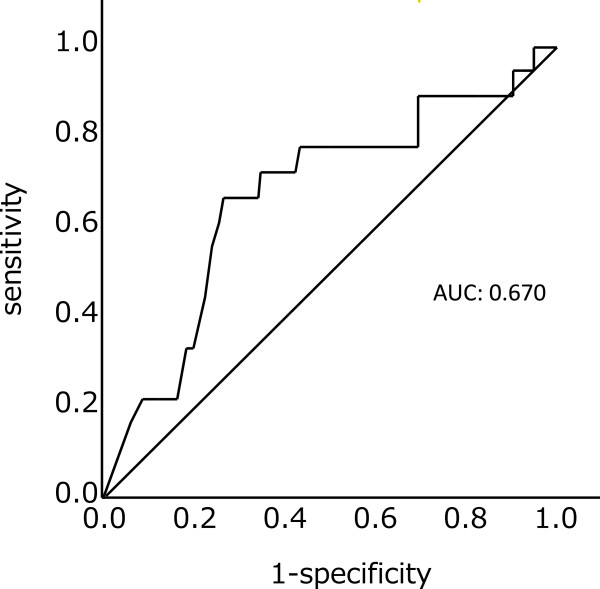
**Receiver operating characteristic (ROC) curve analysis of preoperative CA19-9 for prediction of five-year survival of patients with pancreatic carcinoma** An ROC curve demonstrated that a preoperative serum CA19-9 level of 40 U/mL was the optimal cutoff point. The area under the curve (AUC) was 0.670.

The median preoperative serum CA19-9 level of the 147 patients was 122 U/mL. An ROC curve demonstrated that a preoperative serum CA19-9 level of 40 U/mL was the optimal cutoff point for five-year survival, with a sensitivity of 66.7% and a specificity of 73.6%. The area under the curve (AUC) was 0.670 (Figure 1).

**Table 1:** **Patient characteristics**

Characteristics	Number
Gender	
Male	85
Female	62
Age (years)	
Median (range)	67 (33 to 85)
Tumor location	
Head	97
Body/tail	50
Tumor size (cm)	
Median (range)	3.4 (1.2 to 18)
Surgery	
Pancreatoduodenectomy	90
Distal pancreatectomy	49
Total pancreatectomy	8
Histologic differentiation	
Grade 1	33
Grade 2	80
Grade 3	24
Grade 4	10
UICC stage **Union for International Cancer Control**	
IA	1
IB	7
IIA	46
IIB	71
III	3
IV	19
Positive lymph node	
N0	59
N1	88
Resection status	
R0	72
R1	34
R2	41

Of the 147 patients, 18 patients (12.2%) survived more than five years after surgery without disease recurrence. The median age of five-year survivors (13 men, five women) was 65 years (range: 46 to 76). The distribution of the tumor stages according to The UICC TNM Classification (UICC (Union for International Cancer Control) Sixth) was: stage IA (n = 1; 5.6%); IB (n = 2; 11.1%); IIA (n = 6; 33.3%); IIB (n = 9; 50%); and stage III or IV (none). The pancreatic resections were standard pancreaticoduodenectomy in 12 patients and distal pancreatectomy in six patients. The median tumor size was 32 mm (range: 12 to 55), including five patients (27.8%) with tumor diameters of 20 mm or less. Eight patients were positive for lymph node metastasis, and all patients with positive lymph nodes had within two positive lymph nodes. Portal vein resection was performed in two patients, and R0 surgery was performed in 14 (77.8%) patients. Tumor recurrences beyond five years after surgery were observed in four patients. The longest time for recurrence was 8.4 years after surgery (Table 2).

**Table 2:** **Characteristics of five-year survivors without disease recurrence**

Age/gender	CA19-9 (U/mL)	Tumor location	Tumor size (mm)	UICC stage	TNM-N	Number of lymph node metastasis	Surgery	R	Outcome (month)	Recurrence
71/F	2	Pb	16	IA	0	0	DP	0	68/A	None
76/F	65	Ph	30	IIB	1	2	PD	0	69/D	None
72/M	597	Pt	40	IIB	1	2	DP	1	73/D	Dissemination
57/M	611	Ph	42	IIB	0	0	PD with PV	0	74/A	None
73/F	3221	Pbt	32	IIB	1	2	DP	0	78/A	None
76/M	21	Ph	20	IIA	0	0	PD	0	79/D	None
62/M	103	Ph	40	IIB	1	2	PD	0	85/D	Lymph node
61/M	29	Ph	12	IIA	0	0	PD	1	86/A	None
46/M	31	Ph	40	IIA	0	0	PD	0	89/D	Liver/local
68/F	2381	Ph	55	IIA	0	0	PD	0	92/A	None
73/M	39	Pb	38	IIB	1	1	DP	0	100/A	None
60/F	34	Ph	50	IIB	1	2	PD	0	108/A	None
70/M	29	Ph	32	IB	0	0	PD	0	111/A	None
57/M	20	Ph	30	IIB	1	2	PD with PV	1	122/D	Lung/skin
56/M	4.1	Ph	20	IIA	0	0	PD	0	129/D	None
57/M	30	Pb	30	IB	0	0	DP	0	144/A	None
57/M	7	Pb	35	IIA	0	0	DP	0	145/A	None
70/M	2	Ph	15	IIB	1	1	PD	1	170/A	None

A, alive; D, dead; DP, distal pancreatectomy; Pb, body of the pancreas; Pbt, body and tail of the pancreas; PD, pancreaticoduodenectomy; Ph, head of the pancreas; Pt, tail of the pancreas; PV, portal vein resection; R, resection status.

Table 3 shows the results of the univariate analysis of the factors affecting five-year survival after pancreatectomy in the 147 patients. Sex, age, tumor location, tumor size, histologic differentiation, T classification, N classification, adjuvant therapy, S, RP, CH, LY, V, portal vein resection, and IORT were evaluated, but were not significant on univariate analysis. Significant associations with five-year survival were observed for number of lymph node metastases being two or less (*P* = 0.014), a preoperative serum CA19-9 level cutoff of 40 U/mL (*P* = 0.0018), the absence of NE (P = 0.028), and undergoing an R0 resection (*P* = 0.011).

**Table 3:** **Univariate analysis of factors affecting five-year survival after pancreatectomy for pancreatic adenocarcinoma**

Characteristics	Short-term survivors (n = 129)	Five-year survivors (n = 18)	***P*** value
Gender			
Male	72	13	0.21
Female	57	5	
Age (years)			
Median (range)	67 (33-85)	65 (46-76)	0.28
Tumor location			
Head	85	12	1
Body/tail	44	6	
Tumor size (cm)			
Median (range)	3.5 (1.2-18)	3.2 (1.2-5.5)	0.52
≤2 cm	14	5	0.06
>2 cm	115	13	
Histologic differentiation			
Grade 1-2	102	11	0.13
Grade 3-4	27	7	
T classification			
T1-T2	14	3	0.44
T3-T4	115	15	
N classification			
N0	49	10	0.2
N1	80	8	
Number of resected lymph nodes			
Median (range)	25 (2100)	17 (258)	0.51
Number of lymph node metastasis			
≤2	95	18	0.01
≥3	34	0	
CA19-9 level (U/mL)			
Median (range)	172 (223009)	30.5 (23221)	0.02
≤40	34	12	0
>40	94	6	
Resection margin			
R0	58	14	0.01
R1-R2	71	4	
Adjuvant therapy			
Yes	19	1	0.47
No	110	17	
Serosal invasion			
Present	81	10	0.61
Absent	48	8	
Retroperitoneal invasion			
Present	91	11	0.58
Absent	36	6	
Intrapancreatic common bile duct invasion			
Present	61	7	0.62
Absent	68	11	
Lymphatic permeation			
Present	113	16	1
Absent	16	2	
Vascular permeation			
Present	63	8	0.8
Absent	66	10	
Intrapancreatic nerve invasion			
Present	118	13	0.03
Absent	13	5	
Portal vein resection			
Present	31	2	0.37
Absent	98	16	
Intraoperative radiation therapy			
Present	11	3	0.38
Absent	118	15	

A logistic regression model adjusted for two or fewer lymph node metastases, a preoperative serum CA19-9 level cutoff of 40 U/mL, resection margin status, and the absence of NE identified the following independent cancer-related predictors of five-year survivors: two or fewer lymph node metastases, (OR: 6.02, 95% CI: 1.08 to 112.98; *P* = 0.0385), CA19-9 ≤ 40 U/mL (OR: 5.02; 95% CI: 1.68 to 16.48; *P* = 0.036), and R0 resection (OR: 3.63; 95% CI: 1.12 to 14.28; *P* = 0.0316) (Table 4).

**Table 4:** **Multivariate analysis of factors affecting five-year survival after pancreatectomy with invasive carcinoma of the pancreas**

Predictors	Odds ratio	95% confidence interval	***P*** value
Number of lymph node metastasis			
≤2	6.02	1.08-112.98	0.0385
≥3	1		
CA-19-9 level (U/mL)			
≤40	5.02	1.68-16.48	0.0036
>40	1		
Resection margin			
R0	3.63	1.12-14.28	0.0316
R1-R2	1		
Intrapancreatic perineural invasion			
Absent	2.72	0.645-10.86	0.1664
Present	1		

**Figure 2:**
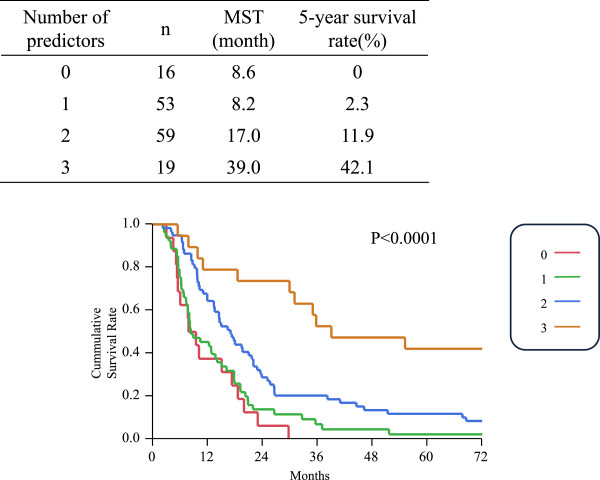
**A combined analysis of the preoperative serum CA19-9 level, R0 resection, and number of lymph node metastases being two or less.** The subgroup of preoperative serum CA19-9 ≤ 40 U/mL and R0 resection, together with number of lymph node metastases ≤2, is associated with a probability of five-year survival of 42.1%. MST, median survival time.

On the basis of the multivariate analysis results, a combined analysis of the preoperative serum CA19-9 level, R0 resection, and number of lymph node metastases being two or less was performed. When each of the three predictors was counted as one point and the points were calculated for all 147 cases, a good stratified survival curve was obtained, showing the longer survival in the higher points: median survival times of three, two, one, and zero points were 39.0, 17.0, 8.2, and 8.6 months, respectively (*P* <0.0001) (Figure 2).

## Discussion

The present study identified three factors (number of lymph node metastases being two or less, preoperative serum CA19-9 level of ≤40 U/mL, and R0 resection), as being related to five-year survival after surgical resection of pancreatic ductal carcinoma. Moreover, the longest survival time was observed in patients who had all of these three factors (Figure 2). To date, prognostic factors for pancreatic carcinoma have been vigorously investigated [4–11]. However, the present study did not simply examine prognostic factors; instead, by analyzing patients who actually achieved five-year survival, factors for five-year survival were more accurately identified.

Tumor size has been considered an important prognostic factor for pancreatic cancer. Large surgical series showed that five-year survival rate (20 to 41%) and median survival time (23 to 38 months) of small pancreatic cancer were better than the five-year survival rate (1 to 20%) and median survival time (10 to 17 months) of large pancreatic cancer [5, 21–24]. In the present study, small pancreatic cancer (≤2 cm) was not a factor related to five-year survival after surgical resection for pancreatic cancer on univariate analysis. About a quarter of patients surviving for more than five years had tumors 2 cm or smaller, suggesting that even patients with a larger tumor can achieve five-year survival.

There are many reports that lymph node metastases are a prognostic factor [4–6]. In addition, the number of positive lymph nodes divided by the total number of lymph nodes evaluated (LNR) has also been reported as a prognostic factor [8]. However, there are few reports limited to the number of lymph nodes. Huebner *et al*. reported new findings on predictive factors for five-year survival when dividing patients into groups with ≤1 versus ≥2 lymph node metastases [25]. In the present study, actual five-year survivors were limited to patients with two or few lymph node metastases. This result suggested that three or more lymph node metastases may mean that lymph node metastases exist outside the area of dissection, or that distal metastases may be present.

In the present study, a CA19-9 cutoff value of 40 U/mL was established using ROC curve analysis as described in the statistical section. The AUC of 0.67 at the cutoff value may be statistically insufficient for sensitivity and specificity, however, it was optimal in our data. Abnormally elevated CA19-9 levels have been reported as a prognostic factor in previous studies, but the cutoff values have ranged widely from 30 to 1,000 U/mL. The normal value for CA19-9 is ≤37 U/mL, and interestingly, a CA19-9 of ≤40 U/mL (near the normal value) was associated with five-year survival in the present study. Waraya *et al*. reported cutoff values of 28 U/mL or 30 U/mL in terms of prognosis, which supports the current results [9]. These results might suggest that long-term survival after surgical resection for pancreatic ductal carcinoma requires that the preoperative CA19-9 level be around the normal range. No other tumor markers, including CEA (Carcinoembryonic antigen) and DUPAN-2, were useful as prognostic indicators (data not shown).

Resection status has also been often reported as a prognostic factor. The present study also found residual tumor status to be an independent predictive factor related to five-year survival. On the other hand, retroperitoneal invasion was not a predictive factor for five-year survival. This important finding means that, even in cases with retroperitoneal invasion, R0 resection is important and hopeful for five-year survival.

In addition, four patients had tumor recurrence beyond five years of follow-up. The longest interval to recurrence was 8.7 years, with lung and skin metastases. Therefore, it should be kept in mind during the follow-up period that recurrences may occur even after five years. Schnelldorfer *et al*. reported that none of the 30 patients who survived beyond 7.8 years had recurrence of disease, and all survived beyond 10 years [6]. Katz *et al*. also reported that late recurrence after five years occurred in seven patients and the latest cancer-related death occurred at 7.6 years [26]. In consideration of our report and the previous reports, survival beyond 10 years might suggest a potential cure.

The CONKO-001 trial [27] reported gemcitabine to be effective as a postoperative adjuvant therapy. This trial reported that treatment with adjuvant gemcitabine led to a 24% improvement in overall survival, with a significant 10.3 percentage point absolute improvement in the five-year overall survival rate (20.7 versus 10.4%), compared with observation alone. Moreover, a recent phase three study compared S-1 and gemcitabine as postoperative adjuvant therapy and reported S-1 to be superior. In this study, in the S-1 therapy group, median relapse-free survival time was 23.2 months, and the two-year relapse-free survival rate was 49% [28]. These results strongly suggest that adjuvant chemotherapy achieved long-term survival after surgical resection. However, in the present study a small number of 20 patients received gemcitabine as adjuvant therapy, resulting in no impact of adjuvant therapy on the survival time (Table 3). Since gemcitabine and S-1 have been recently recognized as standard adjuvant therapies after pancreatectomy for pancreatic cancer with the above mentioned evidences [27, 28], the rate of adjuvant therapy using either of the two drugs has gradually increased in Japan. Nowadays more than 80% of the patients with pancreatic cancer are given gemcitabine or S-1 after pancreatectomy in our institution. With increasing use of adjuvant therapy for pancreatic cancer in the future, five-year survivors would be expected to increase. Although neoadjuvant chemotherapy and neoadjuvant chemoradiotherapy for pancreatic cancer have been investigated for last two decades, their survival benefit has still not been proven [29, 30]. At our institution, neoadjuvant chemoradiotherapy with gemcitabine and S-1 have been used since 2013 for unresectable and borderline resectable pancreatic adenocarcinoma. In our series, some initially unresectable cases have been resectable. But several years are needed to evaluate whether neoadjuvant chemoradiotherapy will become a prognostic factor or not.

Using the predictive model, the subgroup of preoperative serum CA19-9 level cutoff of 40 U/mL and R0 resection, together with number of lymph node metastases being two or less, is associated with a probability of five-year survival of 42.1% (Figure 2). Although long-term survival in this subgroup can be strongly expected, R0 resection and two or fewer lymph node metastases were post-resection parameters. This result suggests that patients not expected to have these factors at the time of preoperative diagnosis should have neoadjuvant treatment or stronger adjuvant chemotherapy.

The limitations of the present study are as follows. This was a retrospective study conducted at a single institution. Approximately 5 to 10% of the general population is Lewis antigen A and B-negative, which means that they do not synthesize the CA19-9 antigen and will not have elevated levels, even with pancreatic cancer or other malignancies. In the present series, the data related to Lewis antigens A and B could not be included because of the retrospective nature of the study.

## Conclusions

In conclusion, the present study showed that two or fewer lymph node metastases, a preoperative serum CA19-9 level of 40 U/mL or less, and R0 resection were associated with five-year disease-free survival of patients with pancreatic cancer who underwent surgical resection. Patients with these three factors are expected to have a high five-year survival rate after surgical resection of pancreatic carcinoma.

## Abbreviations

AUC: Area under the curve
CA19-9: Carbohydrate antigen 19-9
CH: Intrapancreatic common bile duct invasion
CI: Confidence interval
IORT: Intraoperative radiation therapy
LY: Lymphatic invasion
NE: Intrapancreatic nerve invasion
OR: Odds ratio
PV: Portal vein invasion
ROC: Receiver operating characteristics
RP: Retroperitoneal tissue invasion
S: Serosal invasion
V: Venous invasion.
